# A 5-year-old girl with a congenital ganglioneuroma diagnosed by fine needle aspiration biopsy: a case report

**DOI:** 10.1186/1742-6413-5-5

**Published:** 2008-03-30

**Authors:** Marco A Ponce-Camacho, Ricardo Diaz de Leon-Medina, Ivett Miranda-Maldonado, Raquel Garza-Guajardo, Jorge Hernandez-Salazar, Oralia Barboza-Quintana

**Affiliations:** 1 Department of Pathology Hospital Universitario "Dr. Jose Eleuterio Gonzalez" Universidad Autonoma de Nuevo Leon, Monterrey, Mexico

## Abstract

**Introduction:**

Ganglioneuroma is a rare, benign, neuroblastic tumor arising mainly from the central or peripheral autonomic nervous system, especially the sympathetic system. The most affected anatomical sites are the posterior mediastinum, retroperitoneum, adrenal gland and head and neck soft tissue. In the current literature, reports of ganglioneuroma diagnosed by fine-needle aspiration and its cytological appearance are scarce.

**Case Presentation:**

A 5-year-old girl presented with a mass in the cervical region since birth. Laboratory routine tests were within normal limits, ultrasonography demonstrated a solid and well-circumscribed lesion in the soft tissues of the cervical region. Fine needle aspiration biopsy was carried out, and the obtained smears showed a mixture of mature ganglion cells and groups of spindle cells suggestive of schwann cell origin. A diagnosis of ganglioneuroma was suggested. Core biopsy and surgical resection confirmed this diagnosis.

**Conclusion:**

Congenital ganglioneuroma of the cervical region is an uncommon soft tissue benign neoplasm of neuroblastic origin, and it should be considered in the differential diagnosis of head and neck pediatric soft tissue tumors. Fine needle aspiration biopsy technique is a reliable method that can be used with confidence when dealing with pediatric soft tissue tumors.

## Introduction

Ganglioneuromas (GN) are benign, neurogenic tumors arising mainly from central or peripheral components of the autonomic nervous system. The location of the cervical region for this tumor has a reported incidence of only 1–5% in the literature.[1] They are rare compared with other benign neurogenic tumors, such as schwannomas and neurofibromas, but they outnumber neuroblastomas along the sympathetic axis.[2] Reports of the cytological appearance of GN are scarce. We present a case of a GN diagnosed by fine needle aspiration biopsy (FNAB) describing the clinical, cytological and histological features, as in other case reports found in the literature.

## Case presentation

A 5-year-old girl was admitted with a mass in the right parapharyngeal region that had been present since birth. On her physical examination, there was a firm, solid, non-tender mass measuring 4 × 3 cm. The overlying skin was normal. Routine laboratory tests were within normal limits. Ultrasonography (US) showed a solid and well circumscribed lesion. FNAB of the mass was performed.

## Cytological findings

Fine needle aspiration of the mass was performed using a 22-gauge needle and a 10 ml disposable syringe and guided by US. Five smears were obtained and fixed in 95% ethanol. One smear was stained using the Diff-Quick technique and evaluated on site for adequacy, and the remaining slides were stained with the Papanicolau method.

**Figure 1:**
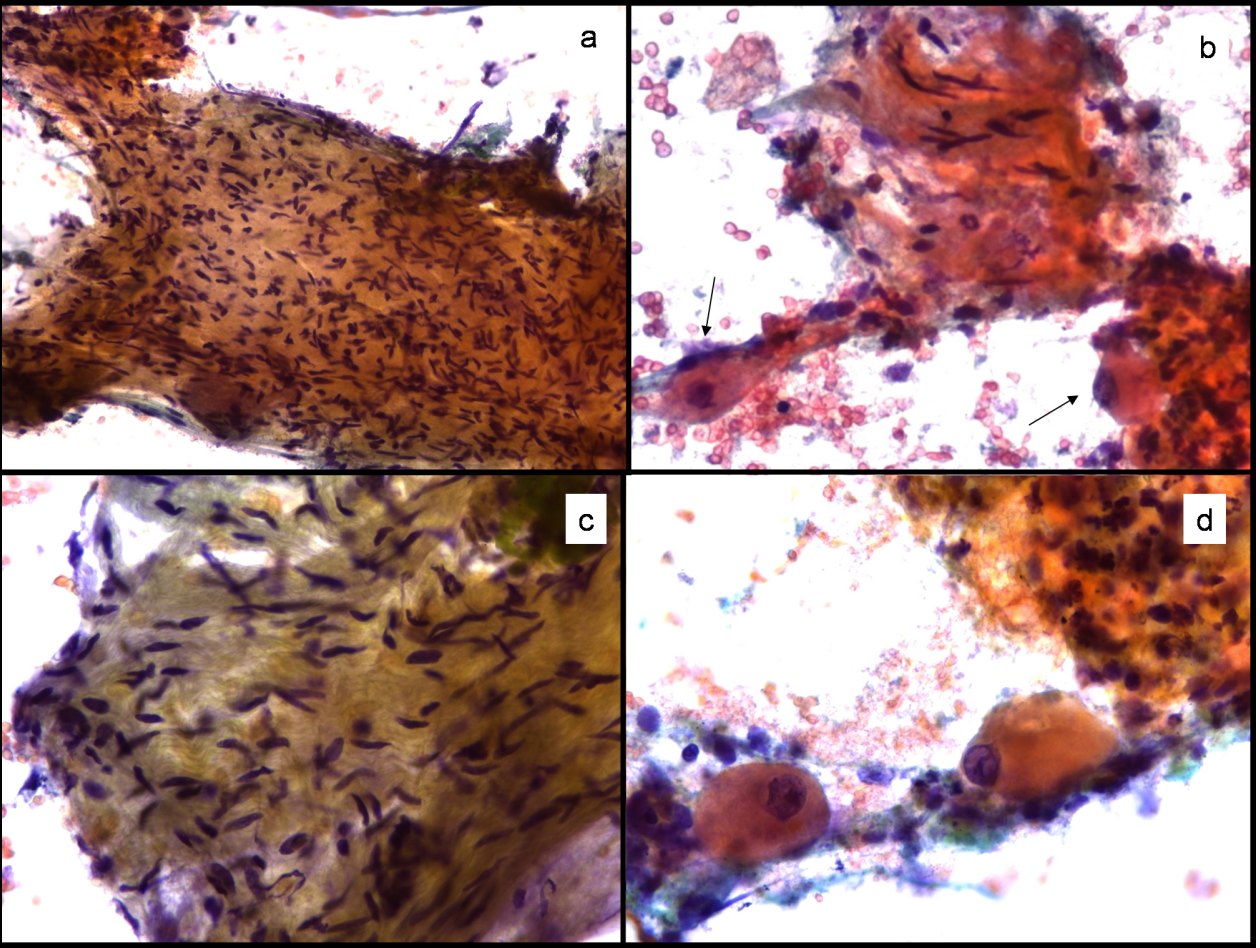
**Cytomorphology**. a) Sheets of closely packed spindle cells. Pap stain 200×. b) Spindle shaped cells intermixed with clusters of mature ganglion cells (arrows). Pap stain 400×. c) Note the slender pinpointed borders of wavy nuclei. Pap stain 400×. d) High power view of oval cells with huge nuclei and conspicuous nucleoli, typical features of a mature ganglion cell. Pap stain 400×.

**Figure 2:**
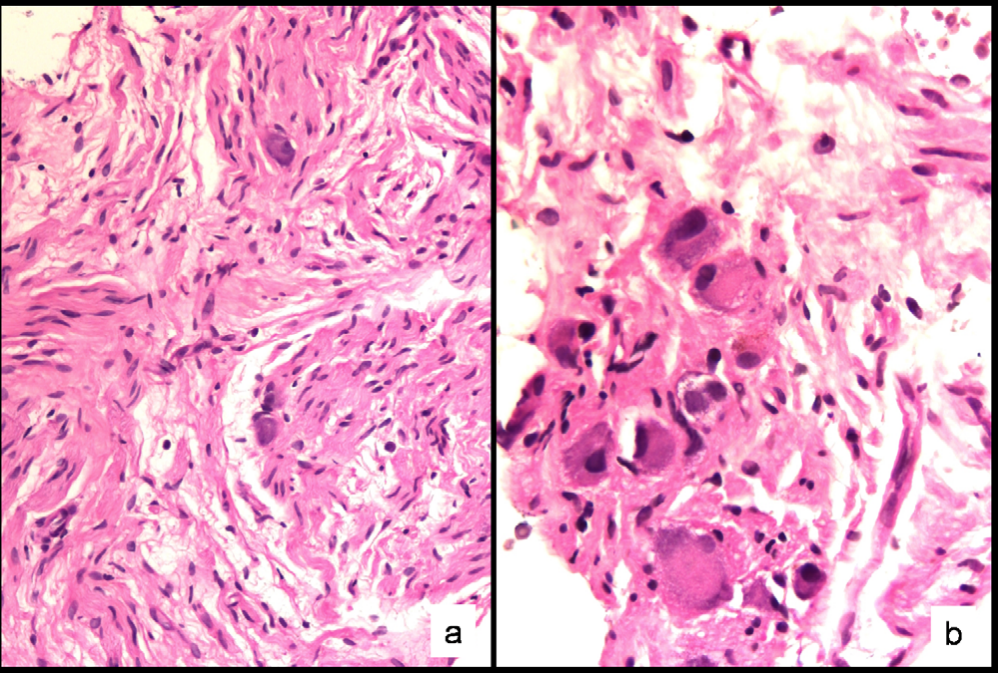
**Biopsy findings**. a) Schwann cells and ganglion cells, the typical features of a ganglioneuroma, are seen in this microphotograph. Hematoxilin & Eosin 200×. b) High power view of a group of mature ganglion cells. Hematoxilin & Eosin 400×.

The aspirates were moderately cellular without a fibrillar or necrotic background. Two different cell populations were observed: the first were spindle-shaped cells, and many of them had wavy nuclei, suggestive of a schwann cell origin. The other cells were large oval polygonal cells with abundant granular cytoplasm. The nuclei were one to three in number and were large, round, vesicular and often eccentrically located; many had a prominent nucleolus (Figure 1). These cells had all the features of ganglion cells. A diagnosis of GN was suggested and a core biopsy was performed. Core biopsy and the resected specimen confirmed a GN (Figure 2). Subsequently, as a result of surgical excision, the patient developed Horner's syndrome [3].

## Discussion

GNs are slow-growing, well-differentiated tumors of the autonomic nervous system, frequently asymptomatic. Clinical manifestations are compression-related symptoms but some patients may develop diarrhea, hypertension, virilization and myasthenia gravis [3]. By definition, GNs have no immature elements (neuroblasts). These neoplasms arise from neural crest neuroblasts that under normal conditions migrate into the adrenal medulla and sympathetic ganglia during fetal development [2]. Solitary GNs most commonly occur in infants and young children, slightly more often in girls than boys, with a female-to-male ratio of about 3:1 [4]. The majority are diagnosed before the patient is 10 years of age. GNs are typically located in the thoracic cavity (60–80%, posterior mediastinum), the abdominal cavity (10–15%, adrenal gland, retroperitoneum, pelvic, sacral and coccygeal sympathetic ganglia, and the organ of Zuckerkandl) and the cervical region (5%) [5,6]. Other less common sites are the middle ear, the parapharynx, the skin, the orbital space and the gastrointestinal tract [7-9]. Ganglioneuroblastomas, the most important differential diagnosis for tumors found in these areas, affects mainly the pediatric age group and are most commonly diagnosed within the first 4 years of life (21 months median age). However, they can be found at any age (some cases are of congenital presentation and others are diagnosed in adults).

In our case, the tumor was found in the cervical region. A literature search revealed only a few case reports in this uncommon site and only one case diagnosed by FNAB in this location [6].

In general, GNs can be easily diagnosed in excision biopsies. The pathologic features of GN are a blend of mature ganglion cells randomly distributed along with a component of spindle cells that corresponds to schwann cells and endoneurial cells. These components can be embedded in a fibromyxoid or fibrillar matrix [10,11]. However, from the cytological point of view, some of these components can make diagnosis difficult since the same previously mentioned cellular components can be seen in other neurogenic neoplasms such as schwannomas and neurofibromas, and an incorrect diagnosis may be made when FNAB fails to yield the ganglion cells [12].

Nerve sheath tumors are benign, mobile, slow-growing masses mainly located in the head and neck and flexor surfaces of the limbs, and less frequently in the mediastinum and retroperitoneum. FNAB smears of nerve sheath tumors are usually low to moderately cellular, containing cohesive elongated or spindle-shaped cells in a background of myxoid material. The cells contain spindle-shaped nuclei which are very slender with pointed ends.

The most striking feature of GN is the presence of mature ganglion cells; mature ganglion cells are characterized as possessing a large nucleus with dispersed chromatin and a conspicuous nucleolus. On the other hand, immature ganglion cells, necrosis, mitosis/karyorrhexis, and the presence of neuropil and calcifications are features of both ganglioneuroblastoma (GNB) and neuroblastoma. In the context of neuroblastic tumors, it is important to achieve a correct diagnosis due to the prognostic and therapeutic implications. Also, it is important to emphasize that ganglioneuroblastomas may only contain small foci of neuroblasts. FNAB or core biopsies can easily miss this immature component. A complete resection of the lesion should be recommended and multisectioning of the whole specimen must be performed, paying particular attention for small foci of hemorrhage or necrosis in order to confirm or rule out the diagnosis of GNB. Clinical data and ancillary procedures such as immunohistochemistry and electron microscopy may contribute to the diagnosis (Table 1) [13,14].

**Table 1:** Differential diagnosis [13, 14]

**Lesion**	**Age**	**Site**	**Cytomorphology**	**IHQ**
Ganglioneuroma	80% < 10 years	Retroperitoneum, posterior mediastinum, pelvis, head and neck	Mature ganglion cells, myxoid stroma, schwann cells, neurites	NSE, Syp, PS-100 (+) CK, Actin (-)
Ganglioneuroblastoma	90% < 5 years	Retroperitoneum, posterior mediastinum	Neuroblasts, rosettes, neuropil, mature ganglion cells, myxoid stroma, schwann cells, neurites	NSE, Syp, PS-100 (+) CK, Actin (-)
Neuroblastoma	90% < 5 years	Retroperitoneum, posterior mediastinum	Neuroblasts, rosettes, neuropil, necrosis	NSE, Syp, PS-100, NbP (+) CK, Actin (-)
Nerve Sheath Tumors	30–50 years	Posterior mediastinum, retroperitoneum, limbs	Cohesive aggregates of spindle cells, myxoid background	NSE, PS-100 (+) CK, Actin (-)
Proliferative fasciitis, Proliferative myositis	All ages	Limbs, head and neck, chest wall	Spindle cells, plump cells ganglion cell-like cells, lack of myxoid background	Vimentin, Actin, PS-100, SMA (+) Syp, CK, NSE (-)

In addition, it is important to recognize that, although only neuroblastic neoplasms disclose ganglion cells, pseudosarcomatous proliferations such as proliferative fasciitis and proliferative myositis may show ganglion cell-like cells [15,16]. These reactive proliferative lesion smears exhibit a mixture of isolated cells and sheets of closely packed spindle cells (fibroblast-like) and giant polyhedral cells with round nuclei and prominent nucleoli (ganglion cell-like cells). The nuclei of spindle cells varies from fusiform to round and plump. The morphologic picture could compromise a correct diagnosis. However, the clinical course of proliferative reactive lesions as well as the immunoprofile of the ganglion cell-like cells can help to elucidate the real origin of the lesion.

## Conclusion

Congenital GN of the cervical region is an uncommon benign soft tissue neoplasm of neuroblastic origin. It should be considered in the differential diagnosis of pediatric soft tissue tumors of the head and neck. The usefulness of FNAB as a rapid, cost-effective and safe diagnostic procedure has been proved, particularly in the pediatric age group where the use of this technique plays an important role in facilitating an appropriate diagnosis and thus an adequate therapeutic approach [17].

## Abbreviations

Fine needle aspiration biopsy (FNAB), ganglioneuroblastoma (GNB), ganglioneuroma (GN), ultrasonography (US), neuron specific enolase (NSE), synaptophysin (Syp), protein S-100 (PS-100), cytokeratin (CK), neuroblastoma protein (Nbp), smooth muscle actin (SMA).

## Competing interests

The author(s) declare that they have no competing interests.

## Authors' contributions

The cytology diagnosis was made by IM-M, RG-G and OB-Q. MAP-C rendered the histopathology report. RDdeL-M and JH-S assisted in the cytology diagnosis. All the authors equally contributed in drafting and designing the manuscript. All the authors read and approved the final manuscript.
